# Microfluidic device enabled quantitative time-lapse microscopic-photography for phenotyping vegetative and reproductive phases in *Fusarium virguliforme*, which is pathogenic to soybean

**DOI:** 10.1038/srep44365

**Published:** 2017-03-15

**Authors:** Jill Marshall, Xuan Qiao, Jordan Baumbach, Jingyu Xie, Liang Dong, Madan K. Bhattacharyya

**Affiliations:** 1 G303 Agronomy Hall, Iowa State University, Ames, IA 50011-1010, USA; 2 2115 Coover Hall, Iowa State University, Ames, IA 50011-1010, USA

## Abstract

Time-lapse microscopic-photography allows in-depth phenotyping of microorganisms. Here we report development of such a system using a microfluidic device, generated from polydimethylsiloxane and glass slide, placed on a motorized stage of a microscope for conducting time-lapse microphotography of multiple observations in 20 channels simultaneously. We have demonstrated the utility of the device in studying growth, germination and sporulation in *Fusarium virguliforme* that causes sudden death syndrome in soybean. To measure the growth differences, we developed a polyamine oxidase *fvpo1* mutant in this fungus that fails to grow in minimal medium containing polyamines as the sole nitrogen source. Using this system, we demonstrated that the conidiospores of the pathogen take an average of five hours to germinate. During sporulation, it takes an average of 10.5 h for a conidiospore to mature and get detached from its conidiophore for the first time. Conidiospores are developed in a single conidiophore one after another. The microfluidic device enabled quantitative time-lapse microphotography reported here should be suitable for screening compounds, peptides, micro-organisms to identify fungitoxic or antimicrobial agents for controlling serious plant pathogens. The device could also be applied in identifying suitable target genes for host-induced gene silencing in pathogens for generating novel disease resistance in crop plants.

Time-lapse microscopic-photography (micro-photography) allows taking images over a period of time for in-depth phenotyping of micro-organisms^1^,^2^,^3^,^4^,^5^,^6^,^7^,^8^,^9^. For example, this technology has been proven useful in studying bacterium-host interactions more accurately than through electron microscopy^9^. Microfluidic technology allows conducting experiments in controlled environments with limited sample volumes ideal for studying responses of micro-organisms to peptides, chemical compounds or *in vitro* transcribed double-stranded RNA (dsRNA) mediated RNAi for identifying target pathogen genes for host-induced gene silencing^10^. Microfluidic chips often contain small channels made of biocompatible silicone (polydimethylsiloxane or PDMS), an optically clear material allowing for easy visualization of biological samples in the channels. Microfluidic devices can also be formed by attaching PDMS channels to the surfaces of other substrates such as glass slide. Based on the design of the channels, desired micro-environmental conditions can be generated on the chip. Microfluidic chips have been used in cell analysis^11^, drug screening^12^,^13^,^14^, plant phenotyping^15^,^16^, and microbial engineering^9^. Recently, microfluidic technology has also been used to study fungi^17^,^18^,^19^,^20^,^21^,^22^,^23^,^24^,^25^,^26^, such as *Pycnoporus cinnabarinus* and *Neuospora crassa*^25^,^26^.

Here we have developed a microfluidic device and showed its application in quantitative phenotyping of growth, germination, and sporulation processes of a fungal plant pathogen with the aid of a stereoscopic microscope. The microfluidic device, containing 20 channels from PDMS and glass slide, was placed on a motorized stage of a stereoscopic microscope for conducting time-lapse photography of multiple observations simultaneously. We have shown that phenotypic data can be acquired through time-lapse microscopic-photography (microphotography) for growth, germination and sporulation in *Fusarium virguliforme*, a fungal pathogen that causes sudden death syndrome in soybean^27^,^28^,^29^,^30^. The quantitative mycelia growth differences were recorded for a polyamine oxidase *fvpo1* mutant and the complemented *fvpo1* mutant that grow differentially in minimal media containing polyamines as the sole nitrogen source. This system allowed us to accurately determine the time taken by conidiospores to germinate. Furthermore, we showed that it takes 10.5 h for a conidiospore to mature and get detached from its conidiophore for the first time. The time-lapse video created in this study also documented that conidiospores are developed in a single conidiophore one after another. The possible applications of this microfluidic device enabled quantitative time-lapse microphotography reported here are also discussed.

## Results

### Microfluidic device enabled time-lapse microphotography

**Figure 1:**
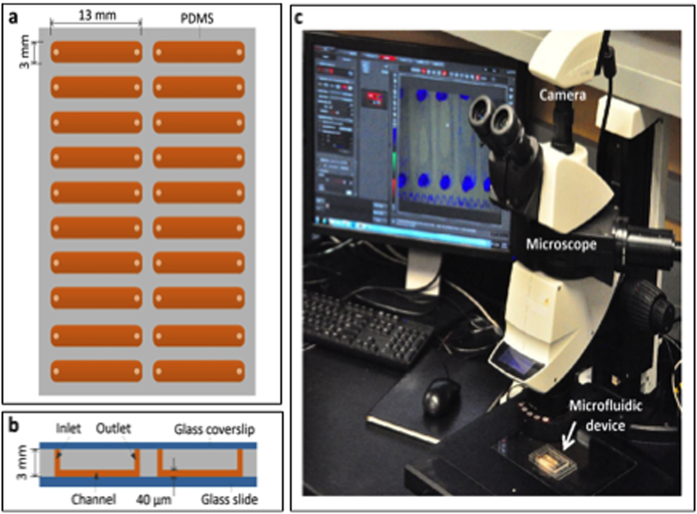
Microfluidic device-enabled time-lapse microphotography system for studying mycelial growth, spore germination and sporulation processes in *F. virguliforme*. (**a**) Microfluidic device (top view) with 20 channels. Each channel is 3 mm wide and 13 mm long. (**b**) Side view of the microfluidic device. Arrow shows a 40 μm deep channel (reddish brown color). The spore suspensions are loaded into each channel through its inlet. The entire device is sealed with a piece of glass (1.2 mm thick) to prevent evaporation. (**c**) Set up of the microfluidic assay. The sealed microfluidic device is placed on the motorized stage (shown by a white arrow) of a Leica stereoscopic microscope.

The microfluidic device was developed from silicone PDMS and glass slide. It contains 20 channels, each with a dimension of 0.04 × 3 × 13 mm^3^ (Fig. 1a and b). The device was manufactured using cost-effective microfabrication techniques described in Methods. The device was placed on a programmable motorized IsoPro XY stage (Leica, Wetzlar, Germany) interfaced with a stereoscopic MZ205 Leica microscope (Leica, Wetzlar, Germany). A digital camera DFC310 FX (Leica, Wetzlar, Germany) was attached to the microscope (Fig. 1c). The motorized IsoPro XY stage was programmed to capture images of selected microscopic fields at a regular time-interval for collecting phenotypic observations. Thus, the microscope allowed taking time-lapse photos of many microscopic fields for quantitative determination of vegetative and reproductive phenotypes of microorganisms, especially fungi or oomycetes.

### Investigation of the germination process in *F. virguliforme*

**Figure 2:**
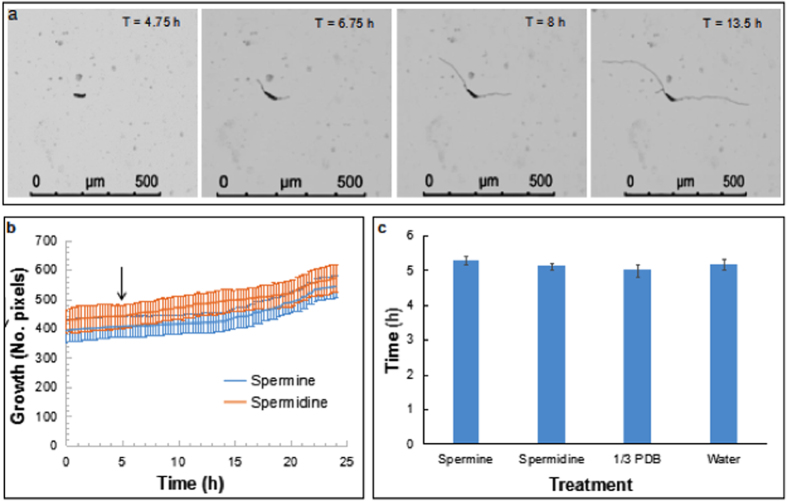
Germination and mycelial growth of *F. virguliforme.* (**a**) Time-lapse microscopic images showing germ tube development, elongation, and mycelial growth. (**b**) Growth of *F. virguliforme* Mont-1 following germination of conidiospores shown in digital pixels. Arrow shows when majority of the spores started to germinate (Table 1). Data are mean ± standard errors of observations collected every 15 min. (**c**) Time taken (h) by the spores to show first sign of germination (Supplementary Table 1).

To investigate the time of germination of conidiospores, spore suspensions in polyamine (PA) media, water, and 1/3 PDB were prepared just before loading into individual channels. Photos were taken in each selected microscopic field every 15 min for up to 24 h (Fig. S1; Fig. 2). We observed that the spores take three and a half to nine and a half hours to germinate (Supplementary Table 1). The average germination time for spores grown in PA media with spermine germinated 5.29 h after being exposed to the media. In the PA media containing spermidine, the spores germinated in 5.11 h following addition to the media. In water, the spores started to germinate in 5.17 h and in 1/3 PDB spores germinate in 4.99 h following suspension to the media (Table 1). We observed no significant difference in the average germination time of spores among the four media (*p* = 0.49). This is consistent with the germination time recorded on a microscope slide. On an average, 35% of spores were germinated five hours after following their suspension in the media (Fig. S2; Supplementary Table 2).

**Figure 3:**
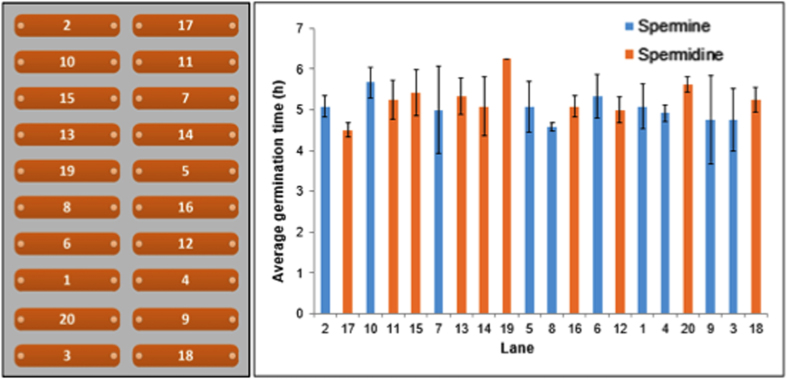
Variation in time taken to show first sign of germination among and within channels. Channel numbers are shown in the left panel. Data are mean and standard errors of three observations in each channel of a typical experiment. Details of the data are presented in Supplementary Table 1. Data are not significantly different (*p* = 0.83 for observations within channel and *p* = 0.37 for observations among 20 channels).

We also used the germination time to determine the variation in experimental conditions across 20 channels. Mean and standard errors were calculated for each of the 20 channels from an experiment with PA media containing either spermine or spermidine and are presented in Fig. 3. Results indicate that in general, germination time of the coniodiospores was uniform with no significant differences in germination time within (*p* = 0.83) and between channels (*p* = 0.36).

### Investigation of the mycelial growth in *F. virguliforme*

**Figure 4:**
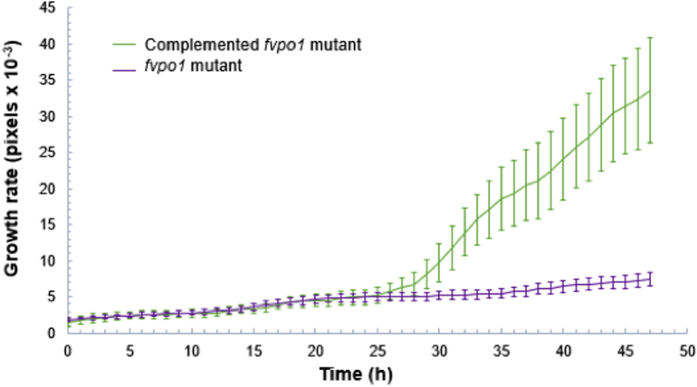
Differential mycelial growth of *Δfvpo1* and complemented *Δfvpo1::FvPO1* isolate in minimal media containing spermine.

To investigate the utility of the device in studying the vegetative growth of the pathogen, we generated a knockout *fvpo1* mutant (*Δfvpo1*) of the *FvPO1* gene encoding a polyamine oxidase that metabolizes polyamines to H_2_O_2_ and nitrogen^31^. A complemented *fvpo1* mutant (*Δfvpo1::FvPO1*) was also generated (Fig. S3). The mutant grows very slowly in minimal PA media containing either spermidine or spermine as the sole nitrogen source. The conidiospores of *Δfvpo1* and *Δfvpo1::FvPO1* were grown in randomly selected channels for up to 48 h. Digital photos of each observation were taken every 30 min and for up to 48 h; the relative mycelial growth of the two isolates in pixels are presented in Fig. 4. As expected, retarded growth was observed in the *Δfvpo1* mutant since it cannot metabolize either spermidine or spermine as efficiently as the *Δfvpo1::FvPO1*. There is a second gene, *FvPO2*, encoding polyamine oxidase in the *F. virguliforme* genome. We speculate that this gene might have been responsible for some growth that we observed in the *Δfvpo1* mutant.

### Investigation of the sporulation process in *F. virguliforme*

**Figure 5:**
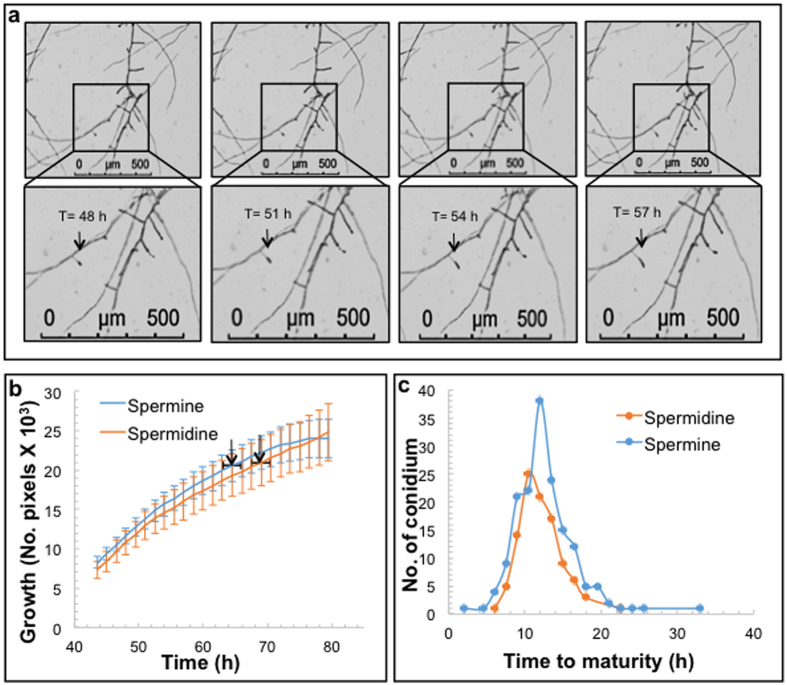
Sporulation in *F. virguliforme* Mont-1. (**a**) Microscopic time-lapse images showing the development of a conidiospore from the first sign of its development on a conidiophore (shown by arrows in the magnified sections of the selected area) grown in PA medium containing spermine. (**b**) Mycelial growth plot showing the time of the first detachment of conidiospores from their conidiophores with arrows (mean ± standard error [horizontal line]). (**c**) Time taken from development to detachment of individual conidiospore.

To study the sporulation in *F. virguliforme*, photos were taken every 1.5 h, from 24 to 120 h, following suspension of the spores in PA media. Collected images were stringed together to generate time-lapse videos (Fig. S4). The videos were analyzed individually to determine the sporulation process. The overall growth patterns of mycelia in two PA media from 44 to 80 h are presented in Fig. 5. We observed that the second conidiospore started to develop immediately following detachment of the first conidiospore from the conidiophore; i.e., the conidiospores were developed on the same conidiophore one after another continuously during the 120 h studied period. On average, the first conidiospore was detached from the conidiophore in 64 h when grown in PA medium containing spermine, and in 69 h when grown in PA medium containing spermidine. The difference in time however was not statistically significant (Table 2). It took *F. virguliforme* 10.7 h to develop a mature conidiospore in PA medium with spermine and 10.5 h in PA medium containing spermidine (Table 2).

## Discussion

In this study, we have investigated a knockout *Δfvpo1* and complemented *Δfvpo1::FvPO1* mutant isolates developed through homologous recombination for studying quantitative differences in mycelial growth (Fig. S3). The mutant lacking the *FvPO1* gene failed to produce polyamine oxidase that metabolizes polyamines such as spermidine or spermine and grew very slowly (Fig. 4). Our data demonstrated that the system is suitable for growing the pathogen and gathering data from 60 locations of 20 channels of the microfluidic device for quantification of the vegetative growth. The pathogen was able to grow in PA media and germinate in around 5 h, which is comparable to 8 h observed for *F. graminearuim*, a closely related fungus, when its spores were suspended in liquid germination medium^32^.

The microfluidic device developed for this study contains 20 channels for replicated experiments. Considering the miniature nature of the device, experimental variations among and within channels were expected to be minimal or nonexistent (Fig. 3). The variations in germination time among and within channels presumably resulted from the biological variation in the spores. Although the pathogen propagates asexually, there are physiological differences among the spores that can affect the time of germination. We observed a range in the time taken by the conidiospores to mature (Fig. 5c). The spores could vary in size and their physiological status, which presumably may have caused some variation in germination time.

PDMS microfluidic devices have been widely used for the development of microfluidic platforms for cell culture and various assays^33^,^34^,^35^,^36^,^37^. Previous studies have shown that cell attachment, proliferation, differentiation, and growth rates could be affected and regulated by many factors, including the wettability (hydrophilicity and hydrophobicity), the mixing ratio of curing agent to base, and the topography and stiffness of PDMS, due to microenvironmental effects^38^,^39^,^40^,^41^. However, as displayed in Fig. 1b, the channels of our device were formed at the bottom of PDMS and attached to the glass slide. Thus, fungi were gown on the glass surface. It is noteworthy that glass is a commonly used material for cell and fungi culture plates and the chemical and mechanical properties of glass surface are stable, so the glass substrate of our microfluidic channels is not expected to influence the growth of fungi even though the top and side walls of the channels were made of PDMS. In our experiment, the surface of PDMS was modified from its original hydrophobic (~110° contact angle for static water drops) to hydrophilic state via oxygen plasma treatment. The surface hydrophobicity of PDMS was partially recovered as indicated by the increase in contact angle from 0 to 77° (Fig. S5). This change did not lead to any noticeable effects on the growth of fungi, because, as mentioned above, the fungi were actually grown on the glass surface of glass, not on the PDMS surface. In fact, when we compared the mycelial growth on glass and PSMS surfaces, no significant difference was observed (Fig. S6).

Time-lapse microscopy was applied in studying growth and sporulation in the bacterium, *Streptomyces coelicolor*^42^. In that study, however, the time-lapse images were collected for one microscopic field at a time. Microfluidic device-enabled quantitative time-lapse microscopic-photography system reported here can capture images from 20 samples simultaneously at a set interval through commands from a computer that are integrated to a digital camera and motorized stage. Thus, this invention will facilitate rapid objective phenotyping of non-motile microorganisms including fungi, oomycetes, bacteria, and nematodes. The device should be suitable also for screening compounds, peptides, micro-organisms to identify fungitoxic or antimicrobial agents for controlling serious plant pathogens. The device could also be applied in identifying suitable target genes for host-induced gene silencing in pathogens for generating novel disease resistance in crop plants.

## Methods

### Microfluidic Device Fabrication

The microfluidic devices used in this study were fabricated using conventional soft lithography^43^. The first step was to make a master mold with photoresist SU-8 (Model SU-8-5; Microchem, Westborough, MA, USA). The mold was made by spin-coating the photoresist on a 3-inch silicon wafer (University Wafer, Boston, MA, USA) at 500 rpm for 5 sec and then at 1,000 rpm for 30 sec. The resulting thickness of the photoresist layer was 35 μm. Subsequently, the wafer was prebaked on a hotplate at 65 °C for 3 min and then at 95 °C for 7 min. The patterns of microfluidic channels were designed by AutoCAD software (Autodesk, San Rafael, CA, USA) and printed on a transparency film photomask (3,600 dpi) using a high-resolution plotter (Fineline Imaging, Colorado Springs, CO, USA). The photomask was put on top of the photoresist and exposed under ultraviolet lights (150 mJ/cm^2^) to form the patterns of microfluidic channels. Next, the patterned photoresist was further baked on a hotplate at 65 °C for 1 min and then at 95 °C for another 3 min. The wafer was then immersed into the developer solution of SU-8 (Microchem, Westborough, MA USA) for 6 min, followed by washing off the wafer with deionized water for 6 min and then drying under nitrogen gas for 3 min. After that, two-parts cure polydimethylsiloxane (PDMS, Sylgard 184; Dow Corning, Midland, MI, USA) precursor solution (weighing ratio of part A:B = 10:1) was mixed and then degassed in a homemade chamber for 30 min under vacuum (10^−4^ Torr). The degassed PDMS solution was poured over the fabricated photoresist mold in a disposable polystyrene Petri dish (100 mm diameter; Sigma-Aldrich, St. Louis, MO, USA) and baked on a hotplate at 80 °C for 1 h. After thermally cured, the PDMS device was peeled off from the master mold. For loading spore suspensions into the microfluidic channels, an inlet and an outlet were manually punched at the two ends of each channel. Next, the PDMS slab was bonded to a glass slide (area: 50 mm × 75 mm; thickness: 0.9 mm; Dow Corning, Midland, MI, USA) by treating with oxygen plasma for 1 min. The device was then baked on a hotplate at 70 °C for 30 min. Finally; another oxygen plasma treatment was given to the whole device for 1 min to make the channels hydrophilic in order to facilitate loading of conidiospores into individual channels.

### Microfluidic Assay

PA medium (volume: 600 μl) containing either spermidine or spermine was used to suspend the conidiospores to a final concentration of around 16 spores per microliter. Approximately 5 μl of spores were added to each of the 20 channels. The spore samples prepared from different genotypes were loaded into the channels at random. Once all channels were loaded, a second glass slide (area: 50 mm × 75 mm; thickness: 0.9 mm; Dow Corning, USA) was sealed on top of the device to minimize any possible evaporation from the channels. Then the device was placed on the motorized stage of the stereomicroscope, which was connected to a computer (Dell Precision 3000, Round Rock, TX, USA). LAS X software installed in the computer was used to program the movement trajectory of the stage and locations of microscopic fields for time-lapse imaging. Three microscopic fields per channel containing one or two spores were marked using the LAS X software (Fig. 1). The same software was used to take images from the selected three microscopic fields from each of the 20 channels simultaneously at a set interval using a digital DFC310 FX, Leica camera (Leica, Wetzlar, Germany).

The images were arranged in the correct sequence and compressed into the Windows Media Video (.wmv) format. A MATLAB code (http://www.memslab.net/uploads/1/1/5/5/11554938/matlab_codes.docx) was used to analyze the images by comparing changes in pixels from image to image to determine how much growth occurred in each marked field. A graph was produced to show the growth changes over time and videos were analyzed to study germination and sporulation processes in *F. virguliforme*.

### *F. virguliforme* isolates

The virulent *F. virguliforme* Mont-1 strain was obtained a single-spore isolate of *F. virguliforme* collected from Illinois in 1991. The isolate was initially obtained on modified Nash and Snyder medium (MNSM; 4) and then maintained on Bilay medium. Before inoculation, cultures of the isolate were grown on potato dextrose agar at room temperature (24 ± 2 °C) in darkness for 14 days. Knockout *Δfvpo1* mutants were created using the homologous recombination protocol described by Pudake and co-workers^44^. Two regions, approximately 1 kb 5′- and 3′-end of the *FvPO1* gene were PCR amplified (Supplemental Table 4) using Cx PFU Turbo Taq polymerase (Agilent Technologies, Santa Clara, CA). The two PCR fragments were cloned into the pRF-HU2 binary vector^44^ using the USER enzyme mix (New England Biolabs, Inc, Ipswich, MA). The resultant plasmid was transformed into *Agrobacterium tumefaciens* EHA-105 strain. Positive EHA-105 colonies were grown overnight at 28 °C in YEP medium (Cold Spring Harbor Protocol, 2006) amended with kanamyacin sulfate (50 μg/ml) and rifamipicin (25 μg/ml). The cultures were used to inoculate IMAS medium^44^ containing kanamycin sulfate (50 μg/μl) and grown till OD_600_ between 0.5 and 0.7. The cultures were then mixed with *F. virguliforme* spores (2 × 10^6^ spores/ml) in a 1:1 ratio. The mixture was then plated on black filter paper (Whatman, GE Healthcare Bio-Sciences, Pittsburgh, PA) layered over IMAS plates. The Filters were transferred to DFM plates^44^ containing hygromycin (150 μg/ml) and cefotaxime (300 μg/ml) after 3 days co-culture. After 3–5 days on the DFM plates, the filters were moved to new DFM plates containing hygromycin (150 μg/ml) and cefotaxime (300 μg/ml). Colonies were selected and screened using PCR and Southern blot analysis^2^ for presence of the hygromycin resistance *Hph* gene and absence of the *FvPO1* gene. For *Δfvpo1* complementation, a fragment containing 3,594 bp including the *FvPO1* 5′-end region, gene and 3′-end region and the 1 kb fragment containing the *FvPO1* 3′-end sequence was amplified using the Cx PFU Turbo Taq polymerase. The fragments were cloned into the pRF-HU2 vector with the hygromycin resistance gene replaced with the geneticin resistance gene using the USER enzyme mix. The resulting plasmid was transformed into *A. tumefaciens* strain EHA-105 and positive colonies were selected and confirmed for stability of the plasmid construct in the *A. tumefaciens* by conducting restriction digestion and gel analysis of the restriction enzyme-digested plasmid. *fvpo1* mutant spores were transformed with the construct following the protocol described above for generating the *fvpo1* mutant. The transformants were selected on geneticin (150 μg/ml) containing plates. The resulting colonies were verified using PCR to demonstrate the presence of the *FvPO1* and geneticin resistance genes. Knockout mutant and complemented *F. virguliforme* isolates were checked for polyamine oxidase activity on minimal media containing spermine and spermidine as the sole nitrogen source to confirm the loss of polyamine oxidase function in the knockout *Δfvpo1* mutant and regain of the activity in a complemented a *Δfvpo1::FvPO1* mutant (Fig. S4).

### Preparation of *F. virguliforme* spores

*F. virguliforme* isolate Mont-1 was grown on solid Bilay medium (0.1% KH_2_PO_4_ [wt/vol], 0.1% KNO_3_ [wt/vol], 0.05% MgSO_4_ [wt/vol], 0.05% KCl [wt/vol], 0.02% starch [wt/vol], 0.02% glucose [wt/vol], and 0.02% sucrose [wt/vol]) and transferred to solid 1/3 PDA medium (0.9% PDA [wt/vol])^45^ a beta-1,3-glucan synthase, is involved in cell wall integrity, hyperosmotic pressure tolerance and conidiation in Metarhizium acridum. Plates were incubated at room temperature in the dark. After 2–3 weeks the plates were washed with 2 mL autoclaved, double distilled water and placed in a clean 1.7 mL Eppendorf tube. The tubes were then centrifuged for 10 sec at maximum speed to pellet the spores. The pellets were then re-suspended in autoclaved, double distilled water (dd H_2_O), repeated two more times to eliminate any leftover nutrients from the 1/3 PDA plates and finally resuspended in 1 ml dd H_2_O. A 1:100 dilution of the spore suspension was prepared in the respective PA medium to count in a hemocytometer. Two readings were taken and averaged to get a more accurate concentration. The spore suspension was diluted in PA medium (1% glucose [wt/vol], 0.02% MgSO_4_ 7H_2_O [wt/vol], 0.3% KH2PO4 [wt/vol], 0.1 mL Trace Elements) containing spermine (500 μM) or spermidine (690 μM), 1/3 PDB (8.8 g potato dextrose broth in 1 L dd H_2_O), or dd H_2_O. The diluted spore suspensions were immediately loaded into individual channels of the microfluidic device.

### Fungal growth comparison

Glass Petri dishes (90 mm diameter) with PDMS were created by pouring 2 ml of PDMS precursor solution (weighing ratio of part A:B = 10:1) into each glass Petri dish and cured at 80 °C for 1 h. Spores were harvested from two-week old *F. virguliforme* culture grown on 1/3 PDA plates. The spores were diluted to a final concentration of 1 × 10^4^ spores per ml in 1/3 PDB. The spore suspension of 12 ml was added to each of six glass Petri dishes or six glass Petri dishes carrying a layer of PDMS. The plates were incubated at room temperature in dark conditions. Fungal mycelia were harvested 48 and 72 hours following incubation. For fungal DNA quantification, 5 ml of the mycelial suspension was centrifuged at full speed and the supernatant was removed. To determine fungal dry weights, 5 ml of the homogenized mycelial suspension from each plate was centrifuged to obtain mycelial pellets. Samples were transferred to pre-weighed 1.7 ml Eppendorf tubes. The samples were washed three times with 1 ml double distilled H_2_O. The samples were placed in an 80 °C oven and dried until constant weight was obtained. Weights were taken after 24 and 48 hours of drying. Student’s t-test was conducted to determine the statistically significant difference in mycelial growth between treatments. Three replications were conducted for each treatment.

## Additional Information

**How to cite this article:** Marshall, J. *et al*. Microfluidic device enabled quantitative time-lapse microscopic-photography for phenotyping vegetative and reproductive phases in *Fusarium virguliforme*, which is pathogenic to soybean. *Sci. Rep.* **7**, 44365; doi: 10.1038/srep44365 (2017).

**Publisher's note:** Springer Nature remains neutral with regard to jurisdictional claims in published maps and institutional affiliations.

## Supplementary Material

Supplementary Information

Supplementary Figure S1

Supplementary Figure S4

## Figures and Tables

**Table 1:** Time taken to for the first visible sign of germination among conidiospores.

Medium	1Visible germ tube (h)
Spermine	5.29 ± 0.11
Spermidine	5.11 ± 0.11
1/3 PDB	4.99 ± 0.17
Water	5.17 ± 0.17

^1^The mean and standard error in each medium were calculated from data presented in Supplemental Table 1. The data are not significantly different (p = 0.49).

**Table 2:** Conidiospore formation in *F. virguliforme.*

PA medium containing	1First conidiospore formed (h)	2Average conidiospore formation time (h)
Spermine	64 ± 2.15	10.74 ± 0.46
Spermidine	69 ± 1.46	10.46 ± 0.42

^1^Data are mean and standard errors of time taken for the first conidiospore to mature, calculated from data provided in Supplementary Table 3. *p *=* *0.078198 from a two tailed T-test.

^2^Data are mean and standard errors of time taken for conidiospores to mature, calculated from the data provided in Supplementary Table 4. *p *=* *0.88197 from a two tailed T-test.
