# Supplementary material for: Microfluidic device enabled quantitative time-lapse microscopic-photography for phenotyping vegetative and reproductive phases in Fusarium virguliforme, which is pathogenic to soybean
Source: Sci Rep. 2017 Mar 15;7:44365. doi: 10.1038/srep44365 (PMC5353701; doi:10.1038/srep44365)
Supplement: Supplementary Information [file srep44365-s1.pdf]

Supplementary Information

Microfluidic device enabled quantitative time-lapse microscopic-photography for phenotyping vegetative and reproductive phases in *Fusarium virguliforme,* which is pathogenic to soybean

Jill Marshall[1*], Xuan Qiao[2*], Jordan Baumbach[1*], Jingyu Xie[2], Liang Dong[2†], and Madan K. Bhattacharyya[1†]

[1]G303 Agronomy Hall, Iowa State University, Ames, IA 50011-1010, USA

[2]2115 Coover Hall, Iowa State University, Ames, IA 50011-1010, USA

[*]These authors contributed equally to this work.

[†]Correspondence and requests for materials should be addressed to M.K.B. mbhattac@iastate.edu or L.D. ldong@iastate.edu

Supplementary Figures

Fig. S1: Time-lapse microscopic-photography of germination of a *F. virguliforme* conidium.

http://www.memslab.net/uploads/1/1/5/5/11554938/supplementary_figure_1.wmv

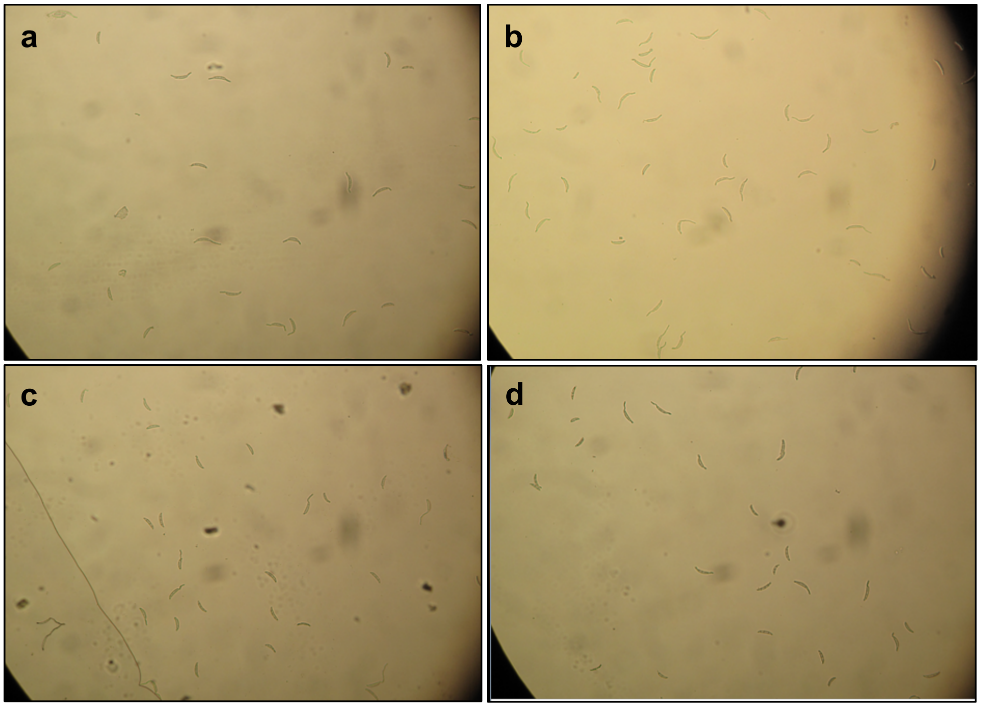

Fig. S2: Germinating spores on microscope slide five hours after being exposed to: (**a**) 1/3 PDB medium (**b**) PA medium with spermidine (**c**) Water (**d**) PA medium with spermine

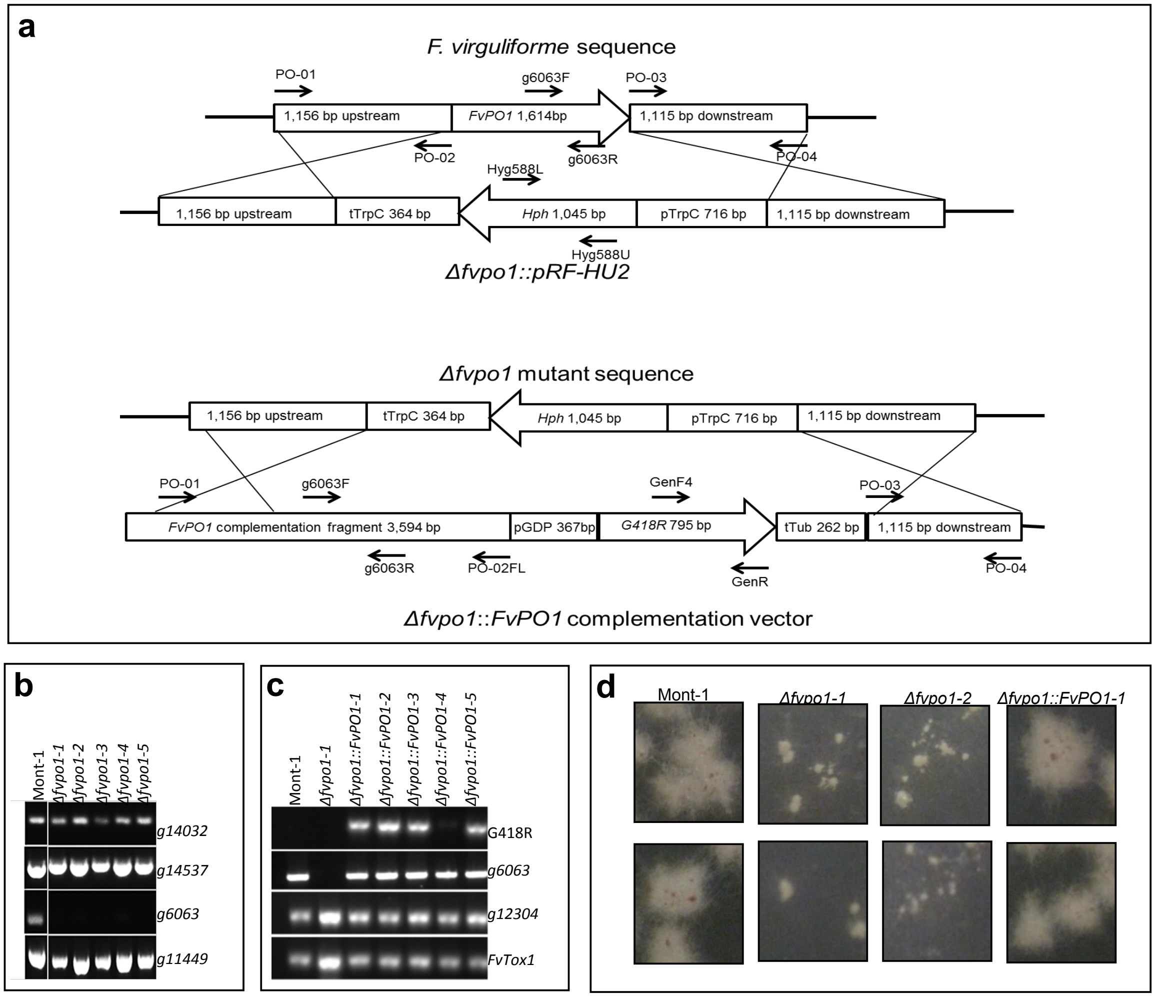

Fig. S3: Construction of *Δfvpo1* knockout and complemented mutants. (**a**) Schematic representation of the strategy for creating both *Δfvpo1* and complemented *Δfvpo1::FvPO1* isolates. Transformations were created using homologous recombination vector pRF-HU2. (**b**) PCR confirming knockout *fvpo1* mutants. Primer location for *g6063* can be found in A. *g14032*, *g14537*, and *g11449* are control genes to show presence of fungal DNA in all samples. (**c**) PCR showing the complementation of the *Δfvpo1-1* mutant. Location of primers for *g6063*, *G418R* (GenF4 and GenR) can be found in A. *g12304* and *FvTox1* were used as controls to show presence of fungal DNA in all samples. All primer sequences can be found in Supplemental Table 4. (**d**) Lack of growth of *fvpo1* in a PA medium. Growth of conidial spores of *Δfvpo1-1, Δfvpo1-2, Δfvpo1-1::FvPO1*, and Mont-1 on PA medium containing spermine as the sole nitrogen source. Reduced growth of *Δfvpo1-1* and *Δfvpo1-2* in a PA medium confirmed the decreased polyamine oxidase activity in these mutants due to loss of the *FvPO1* gene among these knockout mutants. Complemented *Δfvpo1::FvPO1* isolate regained the polyamine oxidase activity and able to grow in a PA medium as the wild-type Mont-1 isolate.

Fig. S4: Time-lapse microscopic-photography of sporulation in *F. virguliforme*
http://www.memslab.net/uploads/1/1/5/5/11554938/supplemental_figure_2.wmv

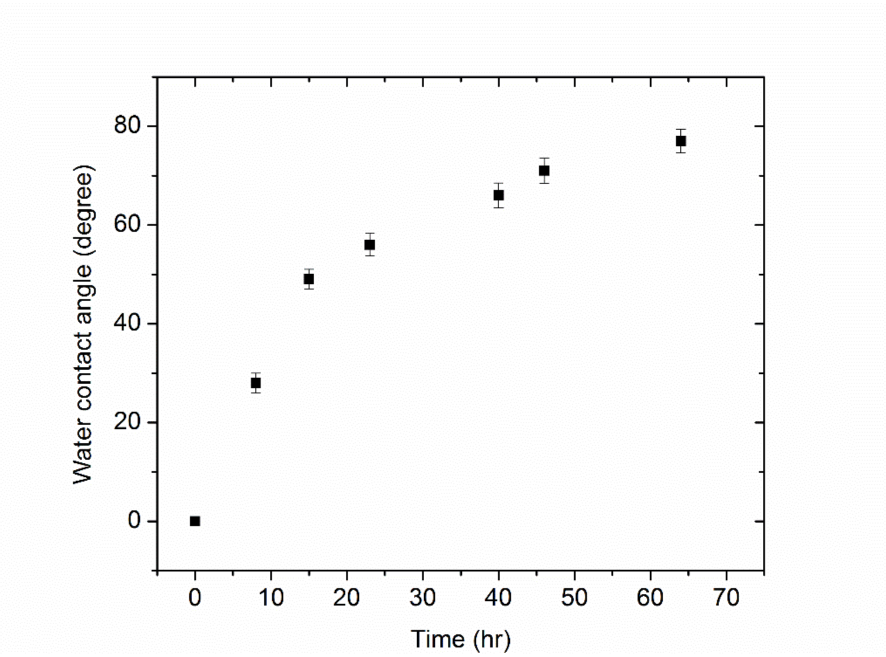

Fig. S5: Water contact angle of PDMS surface as a function of time. The spherical droplets used for the water contact angle measurements were appropriately 1-mm-diameter across. The weighing ratio of base to curing agent of PDMS precursor solution was 10:1. The curing temperature was 80 ºC. The curing time was 1 h.

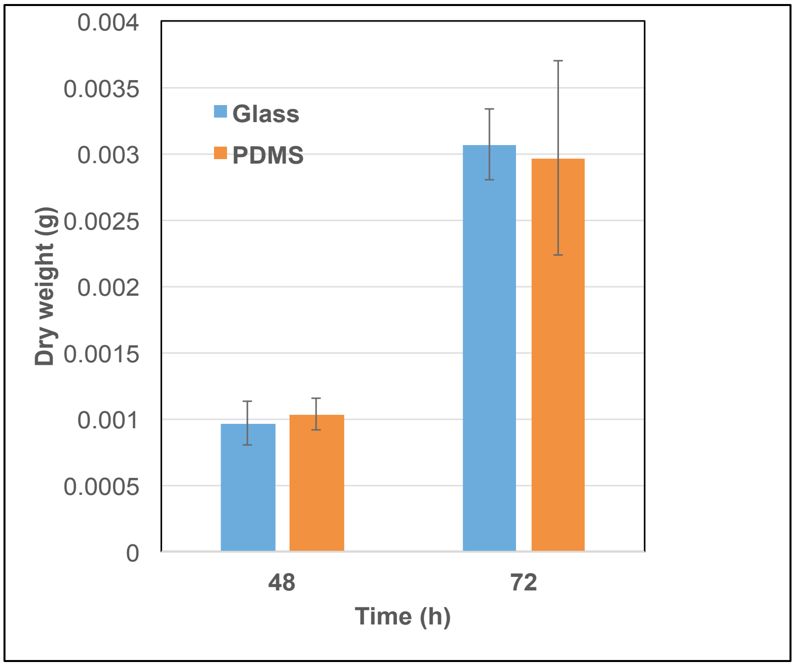

Fig. S6. The graph shows the growth of the fungus on glass plates and glass plates with PDMS. The weight is based on fungal dry weight. Based on a students t-test no significant difference between fungal weights on glass plates and plates with PDMS after 48 hours or 72 hours (p= 0.37 and 0.26, respectively) was observed.

Supplementary Tables

Supplementary Table 1. Time taken to observe the first visible sign of germination.

Observation number	Location on channel	Germination tube visible (h)
PA medium with spermine		
1	163	5.75
2	164	4.25
3	165	5.25
4	166	5.5
5	167	5
6	168	4.75
7	169	4.25
8	170	6
9	171	4
10	172	4.75
11	173	4.75
12	174	5.25
13	175	6
14	176	5
15	177	4.25
16	178	4.5
17	179	5.5
18	180	6
19	181	4
20	182	6.75
21	183	4.25
22	184	4.5
23	185	4.5
24	186	4.75
25	187	4
26	188	6.5
27	189	3.75
28	190	5.5
29	191	5.25
30	192	6.25
31	162	3.75

32	163	5.75
33	164	7
34	165	5.75
35	166	6.25
36	167	5
37	168	5
38	169	5.25
39	170	7
40	171	4.25
41	172	5
42	173	6.75
43	174	5.25
44	175	4.5
45	176	5.25
46	177	6
47	178	5.75
48	180	5
49	181	5.75
50	182	7.5
51	183	5.25
52	185	6.25
53	186	6.25
54	187	5.5
55	188	5.75
56	189	6
57	190	5
58	191	5.25
59	192	4.5
PA medium with spermidine		
1	193	4.5
2	194	5.5
3	195	5.75
4	196	4.5
5	197	5.25
6	198	5.25
7	199	5.25
8	200	4.75
9	201	6

10	202	4.5
11	203	6.25
12	204	4.5
13	205	4.5
14	206	5.75
15	207	6
16	208	5
17	209	5.5
18	210	4.75
19	211	4.5
20	212	4.25
21	213	4.75
22	214	5.5
23	215	4.75
24	216	5.5
25	217	6.25
26	218	6.25
27	220	5.75
28	221	5.5
29	193	5.75
30	194	5.25
31	195	4.25
32	196	5.25
33	197	5
34	198	8.5
35	199	5.75
36	200	5.5
37	201	4.25
38	202	4.75
39	203	5.25
40	204	5.25
41	205	5
42	206	5
43	207	5
44	208	3.75
45	209	4
46	210	4.75
47	211	5
48	212	3.75

49	213	7
50	214	4.25
51	215	5
52	216	4.75
53	217	4
54	218	4.25
55	219	4.5
56	220	7
57	221	4
58	222	4.25
1/3 PDB media		
1	162	4.67
2	163	4.42
3	164	4.67
4	165	5.92
5	166	5.42
6	167	3.67
7	168	5.92
8	169	4.17
9	170	4.92
10	171	5.17
11	172	5.42
12	173	6.17
13	174	5.17
14	175	4.42
15	176	4.42
16	177	4.17
17	178	4.92
18	179	5.17
19	180	4.17
20	181	5.17
21	182	5.42
22	183	4.17
23	184	4.67
24	185	4.67
25	186	4.92
26	187	4.92
27	188	5.67

28	189	5.17
29	190	4.17
30	191	4.17
31	162	3.42
32	163	3.92
33	165	6.92
34	167	9.17
35	168	5.92
36	169	5.67
37	170	3.42
38	171	4.17
39	172	9.42
40	173	3.42
41	174	3.92
42	176	4.17
43	177	3.92
44	178	4.67
45	180	6.17
46	185	3.67
47	186	5.67
48	188	3.42
49	189	5.17
50	190	3.92
51	192	4.17
Water		
1	192	7.17
2	193	4.67
3	194	5.92
4	195	5.42
5	196	4.92
6	197	5.92
7	198	6.17
8	199	5.67
9	200	5.17
10	201	5.42
11	202	5.92
12	203	4.67
13	204	6.67

14	205	4.92
15	206	5.67
16	207	4.92
17	208	5.42
18	209	5.17
19	210	6.17
20	211	4.92
21	212	5.92
22	213	5.67
23	214	5.42
24	215	6.42
25	216	6.17
26	217	5.92
27	218	6.17
28	219	5.42
29	220	4.42
30	193	3.67
31	194	3.42
32	195	3.92
33	196	3.67
34	198	4.67
35	199	3.42
36	200	3.92
37	202	4.42
38	203	3.42
39	204	8.67
40	205	8.17
41	206	3.67
42	207	4.67
43	208	3.17
44	210	5.92
45	211	3.67
46	214	4.17
47	215	4.17
48	216	3.92
49	217	3.42
50	221	3.92

Supplementary Table 2. Percent of spores germinated after five hours in media on glass slide.

Media	Percent germinated
Spermidine	30
Spermine	28
1/3 PDB	30
Water	51
Average	35

Supplementary Table 3. Time taken for the first conidiospore to detach from an individual conidiophore following suspension of spores in liquid PA media.

Observation number	Location on channel	First conidium detaches from conidiophore (h)
PA medium with spermine (12/4/2015)		
1	180	87
2	190	88.5
3	191	85.5
4	192	78
5	193	67.5
6	194	66
7	195	70.5
8	196	66
9	197	76.125
10	201	87
11	202	90
12	204	85.5
13	210	81
14	211	85.5
15	212	63
16	213	64.5
17	214	67.5

18	215	78
PA medium with spermine (12/30/2015)		
19	165	61.5
20	166	54
21	167	60
22	168	57
23	169	64.5
24	171	55.5
25	173	61.5
26	174	57
27	176	58.5
28	177	64.5
29	178	49.5
30	179	48
31	180	49.5
32	181	64.5
33	182	63
34	183	54
35	184	49.5
36	185	52.5
37	187	39
38	188	58.5
39	189	48
40	190	52.5
41	191	46.5
PA medium with spermidine (10/30/2015)		
1	194	72
2	196	76.5
3	198	78
4	199	64.5
5	201	76.5
6	202	76.5
7	203	85.5
8	205	82.5
9	206	63
11	208	66
12	209	72

13	210	64.5
14	211	78
15	213	64.5
16	214	61.5
17	215	75
PA medium with spermidine (12/4/2015)		
18	162	52.5
19	163	64.5
20	164	54
21	165	67.5
22	167	63
23	168	75
24	169	75
25	175	75
26	179	65.8
PA medium with spermidine 12/30/2015		
27	192	61.5
28	196	69
29	197	60
30	198	60
31	202	75
32	203	79.5

Supplementary Table 4. Time taken for an individual conidium to mature and detach from the conidiophore. Note that data in blue font or red font in a microscopic filed indicate the conidium developed from single conidiophores in succession.

Observation Number	Location on channel	Start of spore formation (h)	Spore detaches from conidiophore (h)	Total no. hours to produce a spore
	Spermidine 10/30/2015			
1	164			0
2	171	79.5	91.5	12
3		91.5	105	13.5
4	172			0
5	175			0
6	177	79.5	88.5	9
7		99	114	15
8		103.5	120	16.5
9	183	84	94.5	10.5
10		94.5	106.5	12
11	194	72	82.5	10.5
12		82.5	97.5	15
13		87	100.5	13.5
14		96	103.5	7.5
15		102	115.5	13.5
16	195			0
17	196	75	88.5	13.5
18		79.5	91.5	12
19		90	105	15
20		94.5	111	16.5
21		97.5	111	13.5
22	197			0
23	198	79.5	90	10.5
24		90	103.5	13.5
25		90	99	9
26		91.5	102	10.5
27		91.5	103.5	12
28	199	63	72	9
29		73.5	85.5	12
30		81	99	18

31		103.5	120	16.5
32	200			0
33	201	76.5	87	10.5
34		88.5	102	13.5
35		97.5	114	16.5
36		102	115.5	13.5
37	202	76.5	84	7.5
38		81	88.5	7.5
39		84	94.5	10.5
40		85.5	93	7.5
41		87	93	6
42		91.5	103.5	12
43		94.5	103.5	9
44	203	85.5	99	13.5
45		97.5	106.5	9
46	204			0
47	205	85.5	96	10.5
48		97.5	109.5	12
49	206	70.5	78	7.5
50		72	82.5	10.5
51		73.5	85.5	12
52		90	102	12
53		108	118.5	10.5
54	207	64.5	73.5	9
55		66	76.5	10.5
56		70.5	81	10.5
57		78	87	9
58		79.5	90	10.5
59		87	96	9
60	208	75	87	12
61		75	87	12
62		88.5	99	10.5
63		90	112.5	22.5
64	209	70.5	81	10.5
65		85.5	99	13.5
66		96	114	18
67	210	63	73.5	10.5
68		63	73.5	10.5
69		67.5	81	13.5

70		67.5	81	13.5
71		73.5	84	10.5
72	211	78	90	12
73		82.5	93	10.5
74		99	112.5	13.5
75	212			0
76	213	64.5	73.5	9
77		72	81	9
78		73.5	84	10.5
79		82.5	96	13.5
80	214	60	69	9
81		69	84	15
82		103.5	121.5	18
83	215	66	78	12
84		73.5	88.5	15
85		81	94.5	13.5
86		94.5	111	16.5
Spermidine 12/4/2015				
86	162	51	61.5	10.5
87		61.5	75	13.5
88		61.5	75	13.5
89		78	88.5	10.5
90	163	66	73.5	7.5
91		81	93	12
92		84	94.5	10.5
93		87	102	15
94	164	52.5	61.5	9
95		75.5	88.5	13
96		87	97.5	10.5
97	165	70.5	82.5	12
98		70.5	81	10.5
99		78	87	9
100		84	96	12
101	166	82.5	93	10.5
102		93	108	15
103	167	66	81	15
104		70.5	79.5	9
105		82.5	94.5	12
106		88.5	103.5	15

107	168	90	102	12
108	168	76.5	88.5	12
109	169			0
110	170	79.5	91.5	12
111	171			0
112	172			0
113	173			0
114	174			0
115	175	76.5	88.5	12
116		88.5	105	16.5
117	176			0
118	177			0
119	178			0
120	179			0
Spermidine 12/30/15				
121	192	60	73.5	13.5
122		63	75	12
123		61.5	75	13.5
124		66	79.5	13.5
125		67.5	84	16.5
126	196	64.5	75	10.5
127		66	75	9
128		69	76.5	7.5
129		69	82.5	13.5
130	197	79.5	96	13.5
131		60	72	12
132		75	94.5	19.5
133		76.5	94.5	18
134	198	60	70.5	10.5
135		73.5	88.5	15
136		73.5	87	13.5
137		78	96	18
138	202	75	90	15
139		81	94.5	13.5
140	203	79.5	90	10.5
Spermine 10/30/2015				
1	180	87	99	12
2		90	99	9
3	181	85.5	96	10.5

4	182			0
5	183	81	96	15
6		90	102	12
7	184	85.5	99	13.5
8		96	106.5	10.5
9	185	78	87	9
10		81	90	9
11		85.5	96	10.5
12	186	93	103.5	10.5
13	195	91.5	102	10.5
14	196	90	106.5	16.5
15		99	106.5	7.5
16	199	87	96	9
17		88.5	102	13.5
18		96	108	12
19	201	85.5	97.5	12
20		85.5	97.5	12
21		90	100.5	10.5
22	202	88.5	99	10.5
23	203			0
24	204	70.5	82.5	12
25		73.5	85.5	12
26		76.5	84	7.5
27		87	102	15
28	207	79.5	91.5	12
29		82.5	94.5	12
30		90	103.5	13.5
31	208	60	69	9
32		66	78	12
33		66	78	12
34		69	78	9
35		81	102	21
36	209	66	76.5	10.5
37		66	78	12
38		75	87	12
39		84	103.5	19.5
40	210	67.5	82.5	15
41	211			0
42	212	66	78	12

43	213	70.5	84	13.5
44		84	103.5	19.5
45		85.5	97.5	12
46	214	72	87	15
47		82.5	96	13.5
48	215	78	90	12
49		93	106.5	13.5
50		97	99	2
Spermine 12/30/2015				
51	165	61.5	79.5	18
52		61.5	73.5	12
53		64.5	81	16.5
54		81	93	12
55		82.5	102	19.5
56	166	54	60	6
57		54	69	15
58		55.5	75	19.5
59		73.5	88.5	15
60		93	118.5	25.5
61	167	60	69	9
62		63	69	6
63		64.5	75	10.5
64		67.5	82.5	15
65		67.5	84	16.5
66	168	57	69	12
67		61.5	73.5	12
68		69	79.5	10.5
69		69	79.5	10.5
70		73.5	90	16.5
71	169	64.5	75	10.5
72		76.5	85.5	9
73		82.5	87	4.5
74		94.5	106.5	12
75	171	55.5	66	10.5
76		55.5	79.5	24
77		70.5	82.5	12
78		70.5	79.5	9
79		82.5	99	16.5
80	173	57	67.5	10.5

81		61.5	75	13.5
82		66	82.5	16.5
83		67.5	81	13.5
84		76.5	96	19.5
85	174	57	66	9
86		61.5	76.5	15
87		66	78	12
88		78	90	12
89		94.5	114	19.5
90	176	58.5	73.5	15
91		58.5	73.5	15
92		67.5	78	10.5
93		72	81	9
94		76.5	93	16.5
95	177	64.5	76.5	12
96		66	78	12
97		69	81	12
98		76.5	105	28.5
99		78	91.5	13.5
100	178	45	61.5	16.5
101		49.5	63	13.5
102		55.5	72	16.5
103		63	70.5	7.5
104		76.5	90	13.5
105	179	48	66	18
106		49.5	58.5	9
107		57	70.5	13.5
108		67.5	75	7.5
109		91.5	100.5	9
110	180	63	73.5	10.5
111		73.5	88.5	15
112		75	87	12
113		75	91.5	16.5
114		84	102	18
115	181	64.5	75	10.5
116		66	76.5	10.5
117		78	93	15
118		97.5	111	13.5
119		108	115.5	7.5

120	182	63	85.5	22.5
121		66	82.5	16.5
122		90	106.5	16.5
123	187	39	52.5	13.5
124		45	58.5	13.5
125		52.5	63	10.5
126		57	67.5	10.5
127		66	78	12
128	188	57	67.5	10.5
129		61.5	73.5	12
130		63	73.5	10.5
131		73.5	90	16.5
132		73.5	87	13.5
133	189	48	55.5	7.5
134		57	66	9
135		67.5	84	16.5
136		67.5	79.5	12
137		79.5	97.5	18
138	190	52.5	64.5	12
139		52.5	66	13.5
140		52.5	67.5	15
141		57	67.5	10.5
142		61.5	73.5	12
143	191	46.5	54	7.5
144		48	55.5	7.5
145		57	64.5	7.5
146		58.5	70.5	12
147		61.5	70.5	9

Supplementary Table 5. Primers used in generating *fvpo1* and complemented *fvpo1* mutant.

Primer Name	Sequence
PO-01	ggtcttaaugctggatatgctacattgcgaactc
PO-02	ggcattaauggacgaggtgtgagagctggttcatc
PO-03	ggacttaauggacctgcattgcgatgtatgattg
PO-04	gggtttaauctcgcggacgtggagacatgtaagtg
PO-UU	ggaggacagagtggagaaaggcattg
PO-DD	gaggatctggttcaagaaaccagatc
g6063F	ggtggtcgtatggctcttacagcaac
g6063R	cgtcgttccatgaagaacctcatac
PO-02FL	ggcattaaucagcttgaggcagccaccttcgtacacgatg
Hyg588U	agctgcgccgatggtttctacaa
Hyg588L	gcgcgtctgctgctccatacaa
GenF4	gctgaccgcttcctcgtgctttac
GenR	gaattcactagtaggaacttcggaataggaacttc
